# Exosomal MicroRNA Expression Profiling Analysis of the Effects of Lycium Barbarum Polysaccharide on Gestational Diabetes Mellitus Mice

**DOI:** 10.1155/2020/2953502

**Published:** 2020-07-30

**Authors:** Ya Xiao, Weihao Chen, Ruixue Chen, Anling Luo, Dayi Chen, Qiuer Liang, Tianhao Liu, Xudong Chen, Wei Tan

**Affiliations:** ^1^School of Traditional Chinese Medicine, Jinan University, Guangzhou, China; ^2^The Second Affiliated Hospital, Guangzhou Medical University, Guangzhou, China; ^3^School of Basic Medicine, Jinan University, Guangzhou, China; ^4^Guangdong General Hospital, Guangdong Academy of Medical Sciences, Guangdong Geriatric Institute, Guangzhou, China

## Abstract

**Objective:**

Gestational diabetes mellitus (GDM) is a pathological condition, affecting an increasing number of pregnant women worldwide. Safe and effective treatment for GDM is very important for the public health. In this study, we utilized a high-fat diet-induced GDM model to evaluate the effects of LBP on GDM and examined the changes of exosomal microRNA expression profiling to decipher the potential underlying mechanism of LBP.

**Methods:**

Female C57BL/6J mice were fed a control diet, HFD, or 150 mg/kg LBP-supplemented HFD for 6 weeks before conception and throughout gestation. Oral glucose tolerance test and plasma lipid levels were determined, and liver histopathology was assessed. Sequencing was used to define the microRNA expression profiling of plasma exosomes in the three groups of mice, and protein expression levels of the candidate target genes were analyzed.

**Results:**

LBP significantly relieved glucose intolerance, abnormal plasma lipid levels, and pathomorphological changes of liver histopathology in HFD-induced GDM mice. Moreover, we found that this effect of LBP was mediated by downregulation of the increase of 6 miRNAs (miR-93-3p, miR-188-5p, miR-466k, miR-1188-5p, miR-7001-3p, and miR-7115-5p) and reversing the increase of the protein expression of CPT1A, which is the target gene of miR-188-5p.

**Conclusions:**

Our findings provide novel insights into the biological activities of LBP in the treatment of GDM.

## 1. Introduction

Gestational diabetes mellitus (GDM) is a pathological condition, characterized by glucose intolerance or hyperglycemia resulted from insufficient insulin production or signaling in pregnant women [1]. GDM now affects 2% to 10% of pregnancies and up to 20% of pregnancies in some countries, such as China and India [2, 3]. Not only has GDM been associated with elevated risks of other complications during pregnancy but also poses long-term risks for both mothers and their offspring [4, 5]. Therefore, safe and effective treatment for GDM is very important for the public health.

MicroRNAs (miRNAs) are a class of RNA molecules which play important roles in many biological processes [6]. Extensive studies showed that dysregulation of miRNA expression has been associated with diabetes mellitus [7, 8]. Furthermore, growing evidences indicate that miRNAs are involved in the pathogenesis of GDM [9, 10]. miRNAs show great potential as first trimester biomarkers for GDM as they are located within extracellular vesicles such as exosomes and can be highly stable in body fluids [11]. Exosomes are characterized as 30–100 nm spherical, bilayer lipid vesicles which were generated inside multivesicular endosomes or multivesicular bodies of most nucleated cells and are secreted by exocytosis [12]. Exosomes can mediate cellular communication under both normal and pathological conditions [13]. It has been demonstrated that the concentration of exosomes in plasma increased with gestational age in pregnant women with GDM [14]. Studies have shown that miRNAs within exosomes can be profiled and used as biomarkers for GDM [15, 16]. Consequently, it is necessary to study the role of exosome miRNAs in GDM in-depth. However, the role and miRNA expression profiles of exosomes in GDM remain largely unknown, with studies all specifically focused on human samples [17–23].

*Lycium barbarum* is well known in traditional Chinese herbal medicine and has been widely used as popular functional food for maintaining and promoting the health. Lycium barbarum polysaccharide (LBP) is the major active ingredient isolated from *Lycium barbarum* and possesses a large variety of biological activities. Previous studies have demonstrated that LBP can improve lipid metabolism profiles in animal and human models [24, 25]. Moreover, LBP showed antidiabetic effects in diet-streptozotocin-induced diabetic rats [26] and in patients with type 2 diabetes [27]. However, whether LBP exhibited beneficial effects against GDM is still unclear.

In this study, we utilized a high-fat diet-induced GDM model [28, 29] to evaluate the effects of LBP on GDM and examined the changes of exosomal microRNA expression profiling to decipher the potential underlying mechanism explaining the beneficial role of LBP on GDM.

## 2. Materials and Methods

### 2.1. Animals and Diet

Animal experiments were approved by the Animal Care and Use Committee of Jinan University (approval no. 2017031705005). Animal experiments were performed in the laboratory animal research center of Jinan University. The methods were carried out in accordance with the approved guidelines. Sixty female C57BL/6 J mice at the age of 8 weeks were obtained from Guangdong Medical Laboratory Animal Center (approval no. SCXK (Yue) 2013–0002). All the animals were maintained in a temperature-controlled room (22°C–25°C; 35–55% humidity) with a twelve-hour light/dark cycle. Mice were allowed free access to food and water. Mice were randomly divided into three groups (*n* = 20 per group): control diet (control, D12450B, 10 kcal% fat), high-fat diet (HFD, D12451, 45 kcal% fat), and 150 mg/kg LBP-supplemented high-fat diet (HFD + LBP). LBP was purchased from Ningxia Agricultural and Forestry College. After six-week dietary intervention, female mice were mated with lean male mice. Mating was confirmed by the presence of a vaginal mucous plug the following morning, which represented gestation day (GD) 0.

### 2.2. Body Weight and Lipids

Body weight was recorded on a top-loading balance (Fisher Scientific) before dietary intervention after 6 weeks of HFD and at GD7 and GD16. Lipid levels were measured at gestation day 20. Mice were anesthetized with pentobarbital sodium (60 mg/kg ip), and blood was collected by removing the left eyeball of the mice. Then, the blood samples were rapidly centrifuged at 1000 g at 4°C for 10 min. Plasma levels of triglycerides (TG), total cholesterol (TC), low-density lipoprotein-cholesterol (LDL-C), and high-density lipoprotein-cholesterol (HDL-C) were measured using a multifunctional biochemistry analyzer (Olympus AU2700, Tokyo, Japan).

### 2.3. Oral Glucose Tolerance Test

Glucose tolerance was measured at GD16. Following a 6 h fast, mice were given 2 g/kg glucose solution via oral gavage [30]. Blood samples were collected from the tail at 0, 30, 60, 90, and 120 min, and glucose was measured using a hand-held glucometer (Roche Diabetes Care GmbH, UK).

### 2.4. Histopathology

At GD20, mice livers from all groups were removed and fixed immediately in 10% neutral buffered formalin, dehydrated in gradual ethanol (50%–100%), cleared in xylene, and embedded in paraffin. Sections (4–5 *μ*m thick) were prepared and stained with hematoxylin and eosin (H&E) for morphologic analysis by light microscopy.

### 2.5. Western Blot

Liver tissues were homogenized in radioimmunoprecipitation assay (RIPA) buffer containing protease inhibitor cocktail and phenylmethylsulfonyl fluoride (PMSF). Liver extracts were centrifuged at 13,000 rpm for 10 min, and the supernatant was collected for use in western blot. Protein concentration in supernatants was quantified using the BCA reagent. Aliquots of proteins were analyzed by 12% SDS-PAGE and transferred to the nitrocellulose membrane. The membrane was then immersed in blocking buffer (PBS, 0.1% Tween 20) containing 5% nonfat milk for 1 h and then incubated with primary antibodies HMGCR (ab174830) and CPT1A (CST-12252) (1 : 1000 dilution in blocking buffer) overnight at 4°C. The membranes were further incubated with horseradish peroxidase-conjugated secondary antibodies (1 : 2000; Santa Cruz, USA). Chemiluminescence was detected by the Pierce® ECL western blotting substrate (Thermo Fisher Scientific, USA). The intensity of the bands was quantified using the western blotting detection system Quantity One 4.31 (Bio-Rad, USA).

### 2.6. Exosome Isolation from Plasma and RNA Extraction

Twelve of the pregnant mice in each group were used to perform miRNA sequencing. Exosomes from plasma were isolated using RiboTM Exosome Isolation Reagent (RiboBio, Guangzhou, China) according to the manufacturer's instructions. For exosomal RNA extraction, total RNA was extracted using TRIzol reagent (Invitrogen, Carlsbad, CA). The concentration and purification of RNA were determined by a spectrophotometer (NanoDrop Technologies, Wilmington, DE). The RNA integrity was evaluated by the Agilent 2200 TapeStation (Agilent, Santa Clara, CA). Fixed quantities of RNA of four samples from one group were combined into a single sample. Thus, each group has three biological repeats.

### 2.7. miRNA Library Preparation and Sequencing

Library preparation and sequencing was conducted at RiboBio Co., Ltd. Total RNAs from exosomes were subjected to RNA 3′ adapter ligation and RNA 5′ adapter ligation. Then, the first strand cDNA was synthesized, and PCR amplification was performed. Small RNAs ranging between 18 and 40 nucleotides (nt) were used for library preparation. Finally, sequencing was performed using the Illumina HiSeq 2500 next-generation sequencing platform.

We performed several filtering steps after obtaining the raw reads. Reads that met the following filtering criteria were removed: (1) no 3′ adapter, (2) 5′adapter, (3) excessively long poly A/T sequence, (4) short-sequence reads (length < 18 nt), or (5) low quality. A low-quality read was defined as a read in which >20% of the read bases had a quality value (the error rate of each base sequencing) of ≤20. We also removed reads containing >10% of N bases among the total. Then, further analysis can be conducted. Further analysis identified several categories of small RNAs (miRNA, rRNA, tRNA, snRNA, snoRNA, and piRNA). The annotation of measured small RNAs (rRNA, tRNA, snRNA, and snoRNA) was mapped to Rfam 12.1 (rfam.xfam.org).  The annotation of piRNA was mapped to piRNABank (pirnabank.ibab.ac.in). The remaining sRNAs were mapped to miRBase 21.0 (http://www.mirbase.org) to identify the known miRNAs. miRNA expression levels were estimated by the number of reads per million (RPM).

### 2.8. Bioinformatics Analysis of miRNA

Target genes of the differentially expressed miRNAs with *P* < 0.05 were predicted by four databases, TargetScan, miRDB, miRWalk, and miRTarBase. Gene ontology (GO) functional annotation and Kyoto Encyclopedia of Genes and Genomes (KEGG) pathway enrichment analysis were performed on candidate target genes of the common differentially expressed miRNAs in the three groups using KOBAS 2.0 software.

### 2.9. Real-Time Quantitative RT-PCR Verification of RNA-Seq Data

To further confirm the findings from the RNA-seq analysis, we used the real-time quantitative RT-PCR method to examine the common differentially expressed miRNAs (miR-93-3p, miR-188-5p, miR-466k, miR-1188-5p, miR-7001-3p, and miR-7115-5p) identified in the HFD/control and HFD + LBP/HFD comparisons. Total RNA was extracted from liver tissues of mice in each group using the TRIzol reagent (Invitrogen, CA, USA) according to manufacturer's protocol. The miRNAs' primers were designed by RiboBio Co., Ltd (Guangzhou, China). qRT-PCR was performed with SYBR Premix ExTaqTM II (Takara, Dalian, China) using CFX96 PCR System (Bio-Rad). Relative expressions were normalized to the expression of U6 and calculated using the 2-ΔΔCT method.

### 2.10. Statistical Analysis

All data are expressed as mean ± SEM. One-way analysis of variance (ANOVA) was used to detect statistical significance followed by Tukey post hoc multiple comparisons using software SPSS 22.0. Values of *P* < 0.05 were considered to be significant.

## 3. Results

### 3.1. Effect of LBP on Body Weight in Experiment Mice

As shown in Table 1, body weight of the control group moderately increased throughout the pregnancy, from a mean of 17.9 g before the feeding trial to 20.8 g at the end of HFD, 23.1 g at GD7, and 29.3 g at GD16. In contrast, the HFD group rapidly increased in body weight from a mean of 17.8 g before the feeding trial to 22.4 g at the end of HFD, 25.1 g at GD7, and 30.9 g at GD16. The HFD-treated mice weighed significantly more than the control at the end of HFD and GD7. Body weight of mice in the HFD + LBP group was not significantly different as compared with that of the HFD group.

### 3.2. Effect of LBP on Glucose Intolerance in Experiment Mice

**Figure 1:**
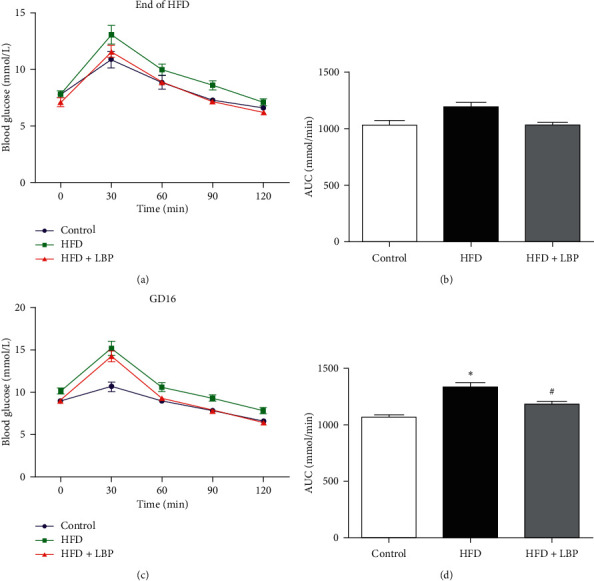
Effect of LBP on glucose intolerance in experiment mice. Glucose tolerance in dams at the end of HFD (a, b) and GD16 (c, d) of mice fed control, HFD, or HFD + LBP diets. ^*∗*^*P* < 0.05 vs. control. ^#^*P* < 0.05 vs. HFD. GD: gestation day; HFD: high-fat diet; LBP: Lycium barbarum polysaccharide.

At the end of HFD and GD16, glucose tolerance was examined by oral glucose tolerance test. HFD feeding tended to increase glucose AUC at the end of HFD, but this effect failed to reach statistical significance (Figures 1(a) and 1(b)). At GD16, the HFD group showed significantly higher fasting blood glucose level than the control group (10.24 ± 0.29 vs. 8.98 ± 0.15). HFD dams exhibited prominent glucose intolerance compared with the control group as indicated by significantly elevated glucose levels at 30, 60, 90, and 120 min and greater AUC (Figures 1(c) and 1(d)). LBP treatment was able to largely alleviate glucose levels at 0, 90, and 120 min and glucose AUC in comparison with the HFD group (Figures 1(c) and 1(d)).

### 3.3. Effect of LBP on Plasma Lipid Levels in Experiment Mice

Table 2 shows significant increases in the levels of plasma TG, TC, and LDL-C and a decrease in the level of plasma HDL-C of the HFD group in comparison with the control group. The administration of LBP prevented increase of TC, TG, and LDL-C and decrease of HDL-C in HFD mice. The results indicated that LBP could attenuate the abnormal changes of lipid profiles in GDM.

### 3.4. Histopathological Analysis

**Figure 2:**
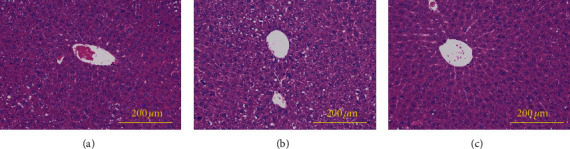
Photomicrograph of a section of the liver of a control diet-fed mouse (a), a HFD-fed mouse (b), and a HFD + LBP diet-fed mouse (c). HFD: high-fat diet; LBP: Lycium barbarum polysaccharide.

As shown in Figure 2, liver sections of mice in the control group had a normal structure in the hepatic cell with distinct nucleus, preserved cytoplasm, and central vein. Livers of HFD-treated mice displayed lymphocytic infiltration, massive fatty degeneration, and loss of cellular boundaries. Mice in the HFD + LBP group exhibited marked improvements in fat deposition and inflammatory cell infiltration, suggesting that LBP can prevent the pathomorphological changes of liver histopathology.

### 3.5. Exosomal Small RNA Transcriptome Profiling

**Figure 3:**
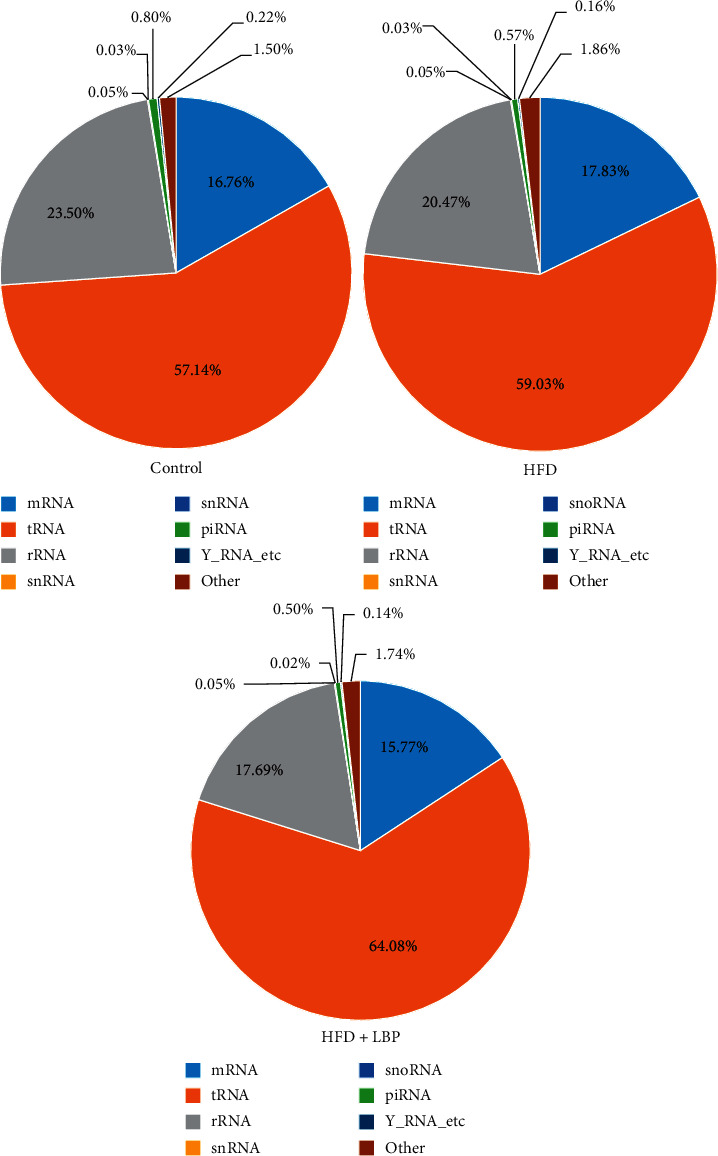
The percentage of small RNA categories in all reads mapped to noncoding RNA databases of mice in the control, HFD, and HFD + LBP groups. HFD: high-fat diet; LBP: Lycium barbarum polysaccharide.

Exosomes have been demonstrated to contain several categories of small RNAs, including miRNA, tRNA, rRNA, snRNA, snoRNA, piRNA, Y_RNA, and unannotated RNAs. The percentages of miRNA in the total small RNA isolated from the control group, HFD group, and HFD + LBP group corresponded to 16.76, 17.83, and 15.77%, respectively (Figure 3). The clean miRNA reads of each sample were mapped to miRBase 21.0. The results of sequence statistics among the samples are listed in Table 3.

### 3.6. Differentially Expressed miRNAs between Samples

Using *P* < 0.05 as the threshold cutoff, the differentially expressed miRNAs between groups were analyzed. Compared with the control group, 18 miRNAs were found to be significantly differentially expressed in the HFD group (7 downregulated and 11 upregulated). Compared with the HFD group, 16 miRNAs were found to be significantly differentially expressed in the HFD + LBP group (6 downregulated and 10 upregulated). As shown in Table 4, 6 common differentially expressed miRNAs (miR-93-3p, miR-188-5p, miR-466k, miR-1188-5p, miR-7001-3p, and miR-7115-5p) were identified in the two comparisons, and LBP treatment can recover the expression levels of 6 miRNAs to normal level.

### 3.7. Enriched GO and KEGG Pathway Analysis

**Figure 4:**
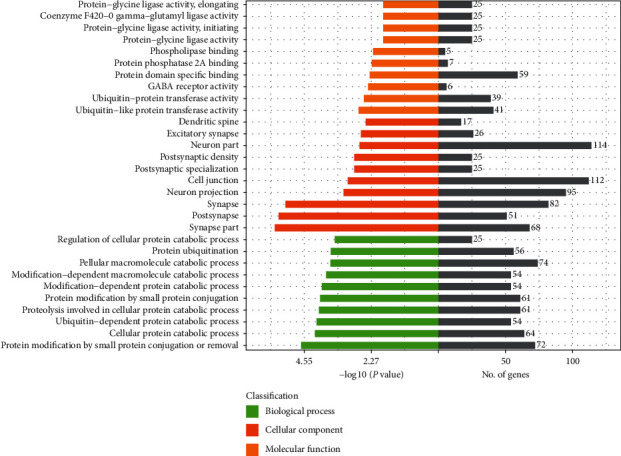
GO enrichment analysis of 6 common differentially expressed miRNAs identified in the two comparisons of HFD/control and HFD + LBP/HFD. HFD: high-fat diet; LBP: Lycium barbarum polysaccharide.

Candidate target genes for 6 common differentially expressed miRNAs in the two comparisons were predicted bioinformatically. GO analysis classified genes by biological process, molecular function, and cellular component. In biological process, genes were mainly enriched in protein modification by small protein conjugation or removal, cellular protein catabolic process, and ubiquitin-dependent protein catabolic process. In the cellular component, genes were mainly enriched in the synapse part, postsynapse, and synapse. In molecular function, genes were mainly enriched in ubiquitin-like protein transferase activity, ubiquitin-protein transferase activity, and GABA receptor activity (Figure 4).

**Figure 5:**
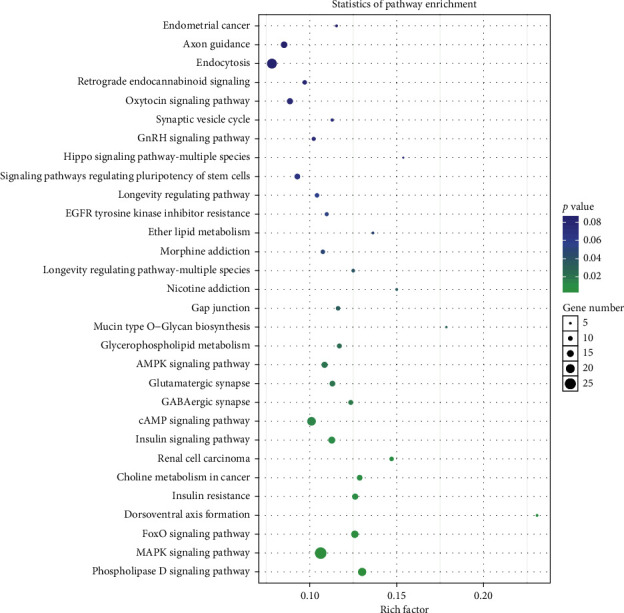
KEGG pathway enrichment analysis of 6 common differentially expressed miRNAs identified in the two comparisons of HFD/control and HFD + LBP/HFD. HFD: high-fat diet; LBP: Lycium barbarum polysaccharide.

KEGG pathway analysis suggested that the genes were evidently enriched in the phospholipase D signaling pathway, MAPK signaling pathway, FoxO signaling pathway, dorsoventral axis formation, insulin resistance, choline metabolism in cancer, renal cell carcinoma, insulin signaling pathway, and cAMP signaling pathway (Figure 5).

### 3.8. Effect of LBP on Common Differentially Expressed miRNAs by qRT-PCR

**Figure 6:**
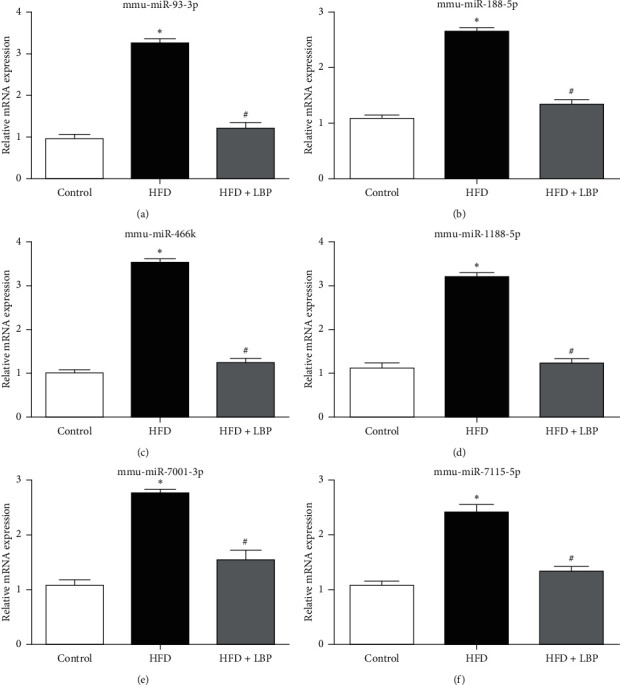
Effect of LBP on common differentially expressed miRNAs by qRT-PCR in livers. Relative mRNA expression of miR-93-3p (a), miR-188-5p (b), miR-466k (c), miR-1188-5p (d), miR-7001-3p (e), and miR-7115-5p (f) in the control, HFD, and HFD + LBP group mice. ^*∗*^*P* < 0.05 vs. control. ^#^*P* < 0.05 vs. HFD. HFD: high-fat diet; LBP: Lycium barbarum polysaccharide.

qRT-PCR was used to validate the expression levels measured by RNA-seq for common differentially expressed miRNAs (miR-93-3p, miR-188-5p, miR-466k, miR-1188-5p, miR-7001-3p, and miR-7115-5p) in livers. As demonstrated in Figure 6, qRT-PCR showed significant increase in the expression of the 6 miRNAs in the HFD group than the control group, and LBP can decrease the expression levels of the 6 miRNAs, which was in correspondence with the findings from the RNA-sequencing analysis.

### 3.9. Effect of LBP on Proteins Associated with Insulin Resistance in Livers

**Figure 7:**
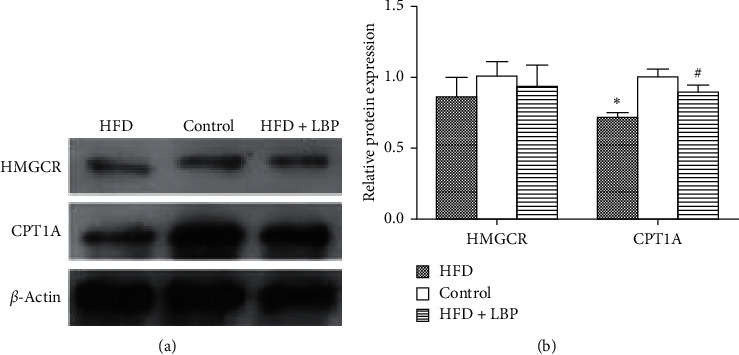
Effect of LBP on proteins associated with insulin resistance in livers. Representative immunoblot (a) and quantification (b) of HMGCR and CPT1A in the control, HFD, and HFD + LBP group mice. ^*∗*^*P* < 0.05 vs. control. ^#^*P* < 0.05 vs. HFD. HFD: high-fat diet; LBP: Lycium barbarum polysaccharide.

We used western blot to determine the effect of LBP on proteins' expression level of the candidate target genes involved in insulin resistance in mice livers. In HFD and HFD + LBP groups, the protein expression of 3-hydroxy-3-methylglutaryl-coenzyme A reductase (HMGCR), which is the target gene of miR-93-3p, was similar to that of the control group (Figure 7(a)). In the HFD group, protein expression of the miR-188-5p target gene carnitine O-palmitoyltransferase 1 (CPT1A) was significantly reduced compared with that of the control group (Figure 7(a)). The decrease of the protein expression of CPT1A in HFD mice was notably reversed by LBP treatment (Figure 7(b)).

## 4. Discussion

The pathogenesis of GDM is complex. Previous studies have demonstrated that genetic predisposition and pregnancy hormones contribute to the development of GDM [31, 32]. However, genetic and hormonal factors are unable to fully explain the pathogenesis of GDM. Environmental factors such as HFD are important constituents of promoting insulin resistance and obesity in pregnancy [33]. Recently, increasing numbers of women are consuming diets high in fats during their pregnancy. As a consequence, being overweight or obese before or throughout pregnancy is a major risk factor for GDM. In the present study, we utilized a HFD (45% kcal fat) in female mice 6 weeks prior to mating to induce weight gain before pregnancy and observed elevated gestational weight gain, impaired glucose tolerance, and abnormal plasma lipid levels, which are clinically similar to human GDM.

Although increasing studies have been conducted on diabetes, very few research studies were specifically tailored towards GDM. LBP has been reported to possess a beneficial effect on diabetes [25, 26]. Given the relative similar cause and symptoms between diabetes and GDM, LBP is highly likely to exhibit similar functions in alleviating GDM. Indeed, out results demonstrated that LBP could remarkably ameliorate maternal glucose intolerance, suggesting that LBP functioned as a potential antidiabetic agent not only under general diabetic condition but also under gestation diabetic condition. The antidiabetic effect of LBP may ascribe to the improvement of abnormal plasma lipid levels and pathomorphological changes of liver histopathology of LBP in HFD-induced GDM mice.

To further explore the mechanism of the beneficial role of LBP on GDM, we examined the changes of exosomal microRNA expression profiling in all three groups of mice. To our knowledge, this is the first study investigating miRNAs in GDM mice. Previous studies investigating miRNAs in GDM all focused on humans. For example, Zhao et al. reported that miR-29a, miR-222, and miR-132 were decreased in serum of women with GDM [17]. Zhu et al. reported that miR-16-5p, miR-17-5p, miR-19a-3p, miR-19b-3p, and miR-20a-5p were increased in plasma of women with GDM [20]. Recently, Almohammadi et al. identified that the expression level of miR-518a-5p, miR-518b, miR-518c, miR-518e, miR-520c-3p, and miR-525-5p in placental exosomes isolated from GDM patients was increased when compared to normal pregnancy [23]. Our results showed that compared with the control group, 18 miRNAs were found to be significantly differentially expressed in the HFD group (7 downregulated and 11 upregulated). However, our results and previous studies share no miRNA in common. Of the 11 upregulated miRNAs, the increase of 6 miRNAs' (miR-93-3p, miR-188-5p, miR-466k, miR-1188-5p, miR-7001-3p, and miR-7115-5p) expression was significantly reversed by LBP. Studies have proved that some of these miRNAs are related to diabetes. miR-93 was related with insulin resistance [34], and it was observed that the plasma level of miR-93-3p was associated with higher risk to develop type 2 diabetes in humans [35]. The expression of miR-188-5p increased in human proximal tubular epithelial (HK-2) cells stimulated by high glucose level [36]. The current state of evidence for the relationship between miR-466k, miR-1188-5p, miR-7001-3p, and miR-7115-5p and diabetes has so far been unknown.

We did the GO and KEGG pathway analysis of the candidate target genes for the 6 miRNAs, and KEGG pathway analysis showed that the genes were evidently enriched in the phospholipase D signaling pathway, MAPK signaling pathway, FoxO signaling pathway, dorsoventral axis formation, insulin resistance, etc. Insulin resistance plays an important role in the development of diabetes. HMGCR and CPT1A, the target gene of miR-93-3p and miR-188-5p, respectively, are closely related not only to insulin resistance but also to abnormal lipid metabolism. Our results showed that GDM mice had hyperlipidemia. Therefore, we choose the insulin resistance-related proteins HMGCR and CPT1A to be verified by western blot. We found that the protein expression of HMGCR, which is the target gene of miR-93-3p, was similar in control and GDM mice, while the protein expression of the miR-188-5p target gene CPT1A was significantly reduced in GDM mice, and it can be notably reversed by LBP treatment. CPT1A can catalyze the entrance of fatty acids into the mitochondria, and it is the rate-limiting enzyme of hepatic fatty acid *β*-oxidation [37]. Our results suggested that LBP had a positive effect on GDM mice which might be due to the enhanced hepatic fatty acid oxidation, which resulted in improved insulin resistance.

## 5. Conclusions

LBP significantly relieved glucose intolerance, abnormal plasma lipid levels, and pathomorphological changes of liver histopathology in HFD-induced GDM mice. Moreover, we found that this effect of LBP was mediated by downregulation of the increase of 6 miRNAs (miR-93-3p, miR-188-5p, miR-466k, miR-1188-5p, miR-7001-3p, and miR-7115-5p) and reversing the increase of the protein expression of CPT1A, which is the target gene of miR-188-5p. The results provide novel insights into the biological activities of LBP in the treatment of GDM.

## Figures and Tables

**Table 1:** Effect of LBP on body weights of mice in the three groups.

Time	Control group	HFD group	HFD + LBP group
Before HFD	17.9 ± 0.2	17.8 ± 0.3	17.6 ± 0.2
End of HFD	20.8 ± 0.2	22.4 ± 0.6^*∗*^	22.7 ± 0.4^*∗*^
GD7	23.1 ± 0.3	25.1 ± 0.5^*∗*^	24.9 ± 0.3^*∗*^
GD16	29.3 ± 0.5	30.9 ± 0.9	31.0 ± 0.4

^*∗*^ *P* < 0.05 vs. control group. GD: gestation day; HFD: high-fat diet; and LBP: Lycium barbarum polysaccharide.

**Table 2:** Effect of LBP on lipid profiles of mice in the three groups.

	Control group	HFD group	HFD + LBP group
Plasma TC (mmol/L)	0.94 ± 0.06	2.03 ± 0.08^*∗*^	1.49 ± 0.08^#^
Plasma TG (mmol/L)	0.53 ± 0.03	0.99 ± 0.06^*∗*^	0.70 ± 0.04^#^
Plasma LDL (mmol/L)	0.14 ± 0.01	0.31 ± 0.02^*∗*^	0.20 ± 0.02^#^
Plasma HDL (mmol/L)	0.45 ± 0.04	0.28 ± 0.02^*∗*^	0.48 ± 0.05^#^

^*∗*^ *P* < 0.05 vs. control group; ^#^*P* < 0.05 vs. HFD group. HFD: high-fat diet; LBP: Lycium barbarum polysaccharide.

**Table 3:** Summary of sequence statistics of the samples.

Group	Total reads	Clean reads	Mapped clean reads	Mapping ratio (%)
Control	15,260,745	13,801,051	10,901,115	79.0
HFD	13,924,992	12,783,536	10,191,842	79.7
HFD + LBP	14,557,650	13,240,453	10,955,990	82.7

HFD: high-fat diet; LBP: Lycium barbarum polysaccharide.

**Table 4:** Differentially expressed miRNAs in the three groups.

miRNA_ID	Log_2_ (fold change)		*P* value	
	HFD/control	HFD + LBP/HFD	HFD/control	HFD + LBP/HFD
mmu-miR-466k	10.414	−10.414	0.011	0.010
mmu-miR-93-3p	10.274	−10.274	0.013	0.013
mmu-miR-7115-5p	10.009	−10.009	0.019	0.018
mmu-miR-1188-5p	9.570	−9.570	0.032	0.030
mmu-miR-7001-3p	9.421	−9.421	0.046	0.039
mmu-miR-188-5p	9.403	−9.403	0.049	0.042
mmu-miR-666-5p	10.124	—	0.001	—
mmu-miR-30c-2-3p	−10.867	8.184	0.002	0.161
mmu-miR-7048-3p	−9.827	8.638	0.004	0.104
mmu-miR-6981-5p	9.998	−1.511	0.019	0.508
mmu-miR-369-5p	−9.812	8.858	0.024	0.099
mmu-miR-6540-5p	−10.048	—	0.025	—
mmu-miR-374b-5p	3.731	—	0.026	—
mmu-miR-709	−3.26	7.534	0.031	0.338
mmu-miR-8097	9.616	−2.344	0.031	0.365
mmu-miR-5129-5p	9.437	−1.336	0.037	0.470
mmu-miR-547-3p	−9.506	6.849	0.046	0.465
mmu-miR-7676-3p	−9.187	—	0.049	—
mmu-miR-7073-5p	—	−10.009	—	0.002
mmu-miR-29b-3p	−1.625	7.143	1	0.004
mmu-miR-190b-5p	3.26	−6.839	0.239	0.019
mmu-miR-378a-5p	—	−9.311	—	0.024
mmu-miR-6952-5p	1.158	−9.586	0.615	0.028
mmu-miR-382-3p	−−	9.597	—	0.035
mmu-miR-10b-3p	−4.143	4.737	0.308	0.036
mmu-miR-6967-3p	−9.202	9.462	0.072	0.040
mmu-miR-6994-3p	—	9.431	—	0.048
mmu-miR-7036b-5p	—	9.588	—	0.049

HFD: high-fat diet; LBP: Lycium barbarum polysaccharide.

## Data Availability

The data used to support the findings of this study are available from the corresponding author on reasonable request.
